# Implementing ethical aspects in the development of a robotic system for nursing care: a qualitative approach

**DOI:** 10.1186/s12912-022-00959-2

**Published:** 2022-07-08

**Authors:** Svenja Nielsen, Sina Langensiepen, Murielle Madi, Maurice Elissen, Astrid Stephan, Gabriele Meyer

**Affiliations:** 1 Institute of Health and Nursing Science, Medical Faculty, Martin Luther University Halle-Wittenberg, Magdeburger Str. 8, 06112 Halle (Saale), Germany; 2 Department of Nursing Science, Uniklinik RWTH Aachen, Pauwelsstraße 30, 52074 Aachen, Germany; 3 Clinical-Surgical Intensive Care Unit, Uniklinik RWTH Aachen, Pauwelsstraße 30, 52074 Aachen, Germany

**Keywords:** Robotics, Nursing, Hospitals, Nursing home, Ethical analysis, User-centered design, Focus groups, Interview

## Abstract

**Background:**

As robotics in nursing care is still in an early explorative research phase, it is not clear which changes robotic systems will ultimately bring about in the long term. According to the approach of “Responsible Research and Innovation”, the research project “PfleKoRo” aims to anticipate and mitigate ethical risks that might be expected when starting to develop a robot. The robot under investigation is intended to be a hands-on support in nursing care in due course. Therefore, the question is which ethical risks and requirements must be considered when developing the robot.

**Methods:**

Guided by the British Standard for the design of robotic systems, ethical risks related to the robot’s use were identified at the outset (Step 1). This was followed by the definition of the requirements needed to mitigate ethical risks (Step 2). Professional nurses, patients and relatives were involved in focus groups and interviews in Step 1. The transcribed interviews and focus groups were then analysed using content analysis. The available literature and expert guidance were taken into account in both steps. Finally, validation and verification methods were defined (Step 3).

**Results:**

Sixteen professional nurses participated in three focus groups. Individual interviews were held with a total of eight patients and relatives. Ethical risks and requirements could be defined in the context of dignity, autonomy, privacy, human relationships and safety in the project. Professional nurses feared most issues relating to safety and that the robot would lead to more workload instead of relief, whereas patients and relatives frequently raised the issue of the staffing ratio. Despite the focus on possible negative consequences, participants also made uncritical or optimistic comments regarding the robot’s use in the future.

**Conclusion:**

Focus groups, individual interviews and existing literature revealed to some extent different ethical issues. Along with identified risks, the results suggest a general open-mindedness of nurses, patients and relatives towards the introduced robot. When investigating the ethical implications of robots for nursing care, one should include multiple perspectives and, in particular, potentially affected individuals.

**Supplementary Information:**

The online version contains supplementary material available at 10.1186/s12912-022-00959-2.

## Background

Robotics may possibly be supportive in future nursing care. According to international interview studies and surveys, nurses and other health professionals judge the main potential of robotics in relieving healthcare professionals both physically and mentally [1–3]. The participants in those studies envisioned that a robot could help by taking on repetitive physical movements as well as repetitive activities like reminding patients and answering recurring questions from people with dementia. Increasing patient safety has also been suggested as a potential benefit of robotics in health care [1, 3]. Earlier evidence suggests robotics in particular as a promising opportunity to increase the mobility and independence of patients [4]. For older people especially, it has been suggested that robots may function as “social facilitators” by stimulating interaction with other people [5]. Furthermore, it has been implied that well-designed systems could be fairer than humans, since robotics is free from prejudices that exist in humans [6].

Despite the opportunities and potential benefits of robots in nursing care, there are also risks. Robotics in nursing is still in a very early explorative research phase and far away from being established in nursing practice. Consequently, it is not clear what changes robotic systems will ultimately bring about for healthcare professionals, patients, informal carers and society in the long term. For this reason, it should be an integral part of research to anticipate possible negative effects of a robot in nursing care right at the beginning of its development process. This is exactly in line with the central ideas of Responsible Research and Innovation (RRI), described as “taking care of the future through collective stewardship of science and innovation in the present”, by Stilgoe et al. [7]. Based on a literature review of 235 articles, four conceptual dimensions making up RRI have been categorized: 1) Inclusion, 2) Anticipation, 3) Responsiveness and 4) Reflexivity [8]. Inclusion aims at involving different stakeholders in the early stages of research while anticipation implies envisioning the future of research and innovation. Responsiveness is closely linked to anticipation as it involves identifying potential risks and reacting accordingly. According to Stilgoe et al. [7], responsiveness further includes being capable of changing direction in response to interested parties or changing circumstances. Lastly, reflexivity means being aware of values and underlying beliefs during research and development.

Several approaches have been suggested to assess the ethical implications of healthcare technologies. However, most of them are primarily intended to help decide about using a technology, the development of which has already been completed. Apart from that, most approaches do not go beyond simply identifying ethical risks. They therefore involve anticipation but not responsiveness, according to RRI, when it comes to “reacting accordingly”. As a result, they are less suitable for actually influencing the development process right from the start. Specifically for the research and innovation in healthcare robotics, it has been argued that there is a “gap” between research in ethics and practice, leading to insufficient consideration of the real problems faced by users. Furthermore, traditional approaches might not be effective to essentially influence the design and consequences of healthcare technologies, such as robots for nursing care [9].

A framework that might overcome shortcomings in robotics research is the BS 8611 “Robots and robotic devices: Guide to the ethical design and application of robots and robotic systems” [10]. The BS 8611 not only aims at giving guidance on the identification of ethical risks, it is also intended to provide guidance in eliminating or reducing risks. The authors also emphasize that risks need to be reviewed with the people affected by the technology. Thus, it is reasonable that the BS 8611 might be suited to implement inclusion, anticipation and responsiveness according to RRI. Other authors also conclude that the BS 8611 could make a positive contribution to RRI in the research area of robots for care [9]. However, it has been criticized that there is no description of how interested parties could actually be involved [9]. Furthermore, as far as the authors of this paper know, empirical insights into how the standard can be applied in nursing and health care have not been published yet.

In the project “PfleKoRo”, a research team of healthcare researchers, physicians, nurses and engineers aims at developing a robotic system based on a model from the KUKA company which is suitable to support nurses when repositioning and holding highly care-dependent and bedridden persons. As part of this project, we aim to explore ethical risks and requirements found to be relevant for the robotic system under investigation, using the BS 8611 as a guiding framework and by involving potential end users.

## Method

### Study design

A qualitative design with focus groups, individual interviews and expert consultation was implemented with the aim of covering multiple perspectives.

The study was approved by the ethics committee of the Medical Faculty of the RWTH Aachen University (EK 427–20) and registered at http://www.germanctr.de (DRKS00028594). Written informed consent was obtained from each participant before inclusion in the study.

### Procedure

Guided by the BS 8611 and modified for our purposes, a three-steps procedure was followed:

1. Identification of ethical risks in the context of the robot.
  For this purpose, ethical risks were extracted from the literature as part of an explorative literature research and included in a list of ethical risks for the project. Members of the research team and a legal expert were asked to validate the list and to add risks if necessary. Potential end users of the robotic system were asked in focus groups and individual interviews about their ethical concerns regarding the system. User-identified concerns were added to the list and re-reviewed by a professional nurse who was a member of the research team. The completed list of ethical risks was transferred to the central risk analysis as per ISO 14971 by a healthcare researcher and an engineer.
2. Formulation of requirements to mitigate identified risks.
  Published recommendations for the ethical design of robotic systems were extracted from the literature and assigned to the risk list as mitigating requirements. The legal expert was again asked for validation and additions or changes to the PfleKoRo system from a legal point of view. Two healthcare researchers and an engineer transferred mitigating ethical requirements to the central requirement list for the robot system in line with Feldhusen & Grote [11].
3. Definition of verification/ validation methods.
  The authors of the BS 8611 suggest several methods for verification and validation, e.g. user validation, software verification or legal assessment. These methods were assigned to the identified risks and ethical requirements.

### Focus groups and individual interviews

#### Recruitment of participants

Convenient sampling method was used to recruit study participants from the University Hospital and a nursing home in the Heinsberg district. The term 'participants' covers participating nurses, relatives and patients. At the University Hospital, a member of the research team informed ward managers about the scope and objectives of the study. Staff nurses as part-time members of the research team working at the University Hospital informed colleagues, patients and relatives personally about the study and passed the contact details of those interested to the coordinators of the interview study. The coordinators contacted potential nurses wishing to participate in the study by email and patients and relatives personally; they also provided more information about the study and arranged appointments for the interviews. The nursing home was involved in the project as a practice partner. The contact person there formed a team of professional nurses who were interested in participating in focus groups for the project and made appointments with the coordinators of the interview study.

Nurses were eligible if they 1) were fully licensed nurses with three years of educational training; 2) had at least one year of professional experience as a nurse and 3) had sufficient knowledge of the German language to take part in an interview. Persons in need of care were recruited if they 1) experienced a need for care in their current situation or in the past 12 months and 2) had sufficient cognitive and German language skills to take part in an individual interview. Relatives were recruited if they 1) belonged to a person in need of care in their current situation or in the past 12 months and 2) had sufficient cognitive and German language skills to take part in an individual interview. 

#### General characteristics of participants

Sixteen out of 31 approached nurses participated in three focus groups (five to six per group); 15 did not attend due to work schedules or for unknown reasons. Four patients and four relatives participated in the individual interviews. The majority of the participants was female (11 of 16 nurses; 7 of 8 patients/ relatives). All the patients were in intensive care units at that time but in a stable health condition. Nine nurses worked in a nursing home and seven in acute hospitals. The participating nurses were between 22 and 61 years old and had 2 to 36 years of working experience. Table 1 displays the participants’ characteristics.

**Table 1:** Characteristics of participants (*n* = 24)

Group of participants	Characteristics	Number
Nurses	Gender	
	Female	11
	Male	5
	Mean age, years (range)	38 (22–61)
	Mean working experience, years (range)	14 (2–36)
	Area of work	
	Nursing home	9
	Intensive Care Unit	5
	Normal hospital ward	2
Patients	Gender	
	Female	3
	Male	1
Relatives	Gender	
	Female	4
	Male	0

#### Data collection

The focus groups and individual interviews were carried out in the German language in May and June 2021. Due to the SARS-CoV-2 pandemic, focus groups with professional nurses were held online using Microsoft Teams. Participants received a document with technical advice and were offered technical assistance in advance. Out of consideration for the situation of intensive care patients and their relatives, individual interviews with these participants were conducted personally at the University Hospital instead of in digital focus groups.

The healthcare researchers SN, SL and MM conducted the focus groups, each with one main moderator and one co-moderator. Some of the participants were already known due to their participation in former studies. In order to address the nurses’ perspectives on ethical issues in relation to the robot under investigation, an interview guide was developed. Questions from the Socratic Approach [12] and HTA Core Model [13], which so far corresponded best to the list of ethical risks, were selected and reduced and modified to five open-ended main questions after a pre-test with four nurses. The question schedule is displayed in Table 2. Before the main questions were asked, the moderators presented an intended application scenario (see additional file 1). Afterwards, participants had the opportunity to become familiar with the frame of questions by means of virtual subgroup discussions (breakout sessions) in Microsoft Teams before the main discussion. Each moderator supervised two to three participants during the breakout session and collected participants’ thoughts on a digital whiteboard that was visible for all participants. Each focus group interview lasted 120 min, including a 20-min break, and was recorded with Microsoft Teams.

**Table 2:** Question schedule for focus groups with nursing professionals

	**Topic**	**Questions**
Opening question	Introduction	Please state your name and the area in which you work as a nurse
Introductory question	Values in care work	What does “good care” involve in your opinion?
Transition question	Affecting values through technology	Have you ever experienced a situation in your job in which you saw these values threatened by the use of technology?
Key questions	Ethical concerns	What undesirable consequences could the use of the robot have?
		How could the robot affect the esteem or reputation of care recipients/ caregivers?
		How could the robot change the relationship between caregivers and those in need of care or the relationship among caregivers?
		How could the robot compromise the privacy of persons in its vicinity?
		How could the robot affect the self-determination/ independence of those in need of care/ nursing professionals?
Final questions	Consolidation	Which of the discussed aspects should we consider most urgently? Please name up to three
		Did you miss any aspects in our discussion? Is there anything else you would like to say?

To address patients and relatives’ perspectives on ethical issues relating to the robot, one open question was integrated into individual interviews for a broader purpose within the project: What negative consequences could the robot have for you/ your relative or for care in general? Participants were shown a short video of the robot under investigation beforehand. A physician and member of the research team conducted the interviews, which lasted between 13 and 26 min and were audio-recorded using a conventional voice recorder.

All focus group and individual interviews were transcribed verbatim and the recordings were deleted afterwards.

#### Data analysis

The transcribed interview material was content-analysed in line with Kuckartz [14] and supported by MAXQDA software. Two healthcare researchers analysed the data together. Initial main codes were derived deductively from the question schedule. For example, the key question “How could the robot compromise the privacy of persons in its vicinity?” was transferred into the main code “dignity”. Further main codes and subcodes were derived inductively from the data. For example, safety issues were not specifically addressed in the question schedule, but were raised by the participants during the discussion. Consequently, the further main code “safety” was formed according to the data. The code system was continuously adapted during the analysis process and discussed with the team. One of the nurses in the team of researchers was involved during the analysis process and validated the final code system with coded text elements. The final code system consists of seven main categories with up to five subcategories each. The codebook with all the subcategories, category definitions and anchor examples is presented in additional file 2.

## Results

### Qualitative content analysis

Seven main categories emerged from the data and are described below, along with quotes from the focus groups and interviews.

#### Main category 1: dignity

In this category the participants’ fears as to how the robot’s use might affect the sense of value or reputation of persons or institutions were analysed. The participants could imagine that those in need of care might feel less worthy and less perceived as a human being when touched by a robot instead of by a human caregiver. One participant justified this with parallels to industry: "The patient might feel being the subject of mass processing because at the moment a robot arm is mainly known as part of the production line in the automobile industry. That is, he would not feel valued or perceived as a person" (Professional Nurse (PN) 1, Focus Group (FG) 3). In this regard, a patient also expressed uncomfortable feelings about “that such a soulless being should do all these movements” (P 4).

The participants argued further that the robot could even frighten those in need of care: "That can sometimes lead to the patient being frightened, when there is suddenly such a device at his bedside" (PN 10, FG 2). This was mentioned with regard to all people in need of care, but in particular for those with cognitive diseases and those who are not generally used to modern technologies.

Furthermore, the possible effects on the reputation of professional caregivers and institutions were discussed. Some participants feared that their work might be less valued, as people might think that human care could be replaced by robots or the work would no longer be so demanding when a robot is used. One participant stated "It reduces our, my sense of value, or affects the whole profession, because it sounds the same as when someone says "Anyone can nurse”” (PN 12, FG 1).

#### Main category 2: autonomy

The participants discussed how the robot might threaten nurses’ and patients’ ability to decide and act independently. On the one hand, they talked about the possibility to decide about the use of the robot. One participating nurse expressed concern about pressure from the employer who might focus on the economic aspect: "If a lot of money has been paid for it, then it ought to be used" (PN 9, FG 1). On the other hand, they indicated that abilities of both nurses and people in need of care might get lost as a consequence of the robot’s regular use. One participant stated that "if a residual ability or resource is there and the resident […] or because the robot takes over, the resources become more limited or are lost completely" (PN 4, FG 1). Concerning the part of nurses, one participant raised the concern that “the technology can very quickly be overestimated and that too much reliance is placed on the technical aspect” (PN 10, FG 2).

#### Main category 3: personal privacy

Discussion under this category revolved around possible negative consequences for the privacy of people in the robot’s environment. Some participants tended to the view that patients and caregivers might feel uncomfortable due to the need of cameras and microphones for the robot to function. They asked themselves what kind of data might pass the robot’s sensors, such as recordings of private conversations or photos of patients’ intimate areas. In this context, a professional nurse thought "One might feel that one is being monitored a bit due to the camera" (PN 11, FG 3).

Further concerns focused on the use of data. Some nurses expressed concerns that data might be used to monitor their work and hold them accountable. One nurse linked this to the relationship with the employer: "Have I got a boss who looks at these recordings […] or is my professionalism being trusted, and the material is not being used" (PN 5, FG 3). Participants asked themselves in general who might have access to the data. One participant asked if “the health insurance companies [would] be able to make use of it or something like that?” (PN 12, FG 1).

#### Main category 4: relationship level

In this category, participants’ thoughts about possible negative effects of the robot on human relationships were analysed. On the one hand, participants considered how contact with the person in need of care could suffer. Participants mainly expressed the view that the contact to the patient might suffer under the robot’s use because patients were unsettled by the robot’s presence or nurses were busy handling the robot. One relative formulated that there might be “less human contact perhaps or fewer conversations, less direct approach, because perhaps the nurse communicates more with the robot at that moment and not with the patient” (Relative (R) 4).

On the other hand, participants thought of possible consequences for the relationship between nurses. Nurses from a clinical setting explained they often work together with colleagues and are therefore worried about less teamwork when working with the robot: "And there is togetherness and teamwork at the bedside when staff help each other to move patients or make the beds, and that would then all be lost" (PN 13, FG 1).

#### Main category 5: safety

Issues of safety were not explicitly addressed by the question schedule but were named initially and spontaneously by participants. As an interface to classical risk analysis, participants mentioned that people in need of care could suffer harm such as skin lesions and pain caused by the robotic end effector when touching the patient. For instance, nurses raised the risk of fractures when moving a patient: "If there is muscular resistance or something, that it doesn't break your bones later on" (PN 14, FG 2).

Participants also mentioned doubts concerning the robot’s abilities and that malfunction or breakdowns could endanger patients. Nurses indicated that another perspective might be missing, as the robot does not have the observation and communication skills that human colleagues do: "Well, if the robot has to turn a patient over […] and there's a wound or something, the nursing professional can't see that, and the robot can't pass it on either, like "Look here, there's a wound” (PN 7, FG 1).

During the discussions, nurses raised further concerns about the robot’s level of autonomy and the possibilities to control it: "But the robot is voice-controlled and then makes his own actions and that is, again, if I'm not stood right next to it, impossible to control it that way “ (PN 14, FG 2).

#### Main category 6: organizational matters

All fears that could not be assigned to the other categories in terms of content were dealt with in this category. The only topic that emerged predominantly not only in the focus groups but also in the individual interviews was the robot’s effects on the staffing ratio which was mentioned here by six out of eight patients and relatives. Participants raised concerns about whether the robot might be counted as a human caregiver rather than as serving as an additional support for the nurses. One relative stated "For nursing in general, I see the danger that the care robot will replace the nursing staff, who will be redundant" (R1).

Another concern of the participants was the question of efficiency. They discussed the fear that the robot might not lead to relief but to more work in operating the robot like in terms of bringing it to the patient, handling during usage, or documentation. One relative furthermore mentioned “the time that is needed to teach the patients, explain everything so that they accept it. […] Probably having to check each morning if it is still working properly and charge it" (R4).

Apart from that, nurses raised the question of liability in the event of mistakes during working with the robot: "If it's really because it's not controlled properly, then I think the liability issue is that the nurse is responsible" (PN 2, FG 2). Finally, issues of distributive justice were mentioned due to limited availability.

#### Main category 7: positive aspects

The focus groups were conducted to identify ethical concerns regarding the robot from the user’s perspective. Accordingly, the moderation and analysis focused on negative aspects that could result from the robot’s use. Nevertheless, many participants mentioned positive effects of the robot or contradicted the negative effects discussed. Regarding the aspect of self-esteem as a nurse, one participant stated: "It can't do holistic nursing. It can do the—the mechanical thing, but not everything else, it can't carry out the whole process" (PN 12, FG 1). In the context of autonomy, one nurse highlighted a possible positive effect of the robot: "I don't need to wait for a colleague, I can perform tasks directly one after the other" (PN 7, FG 1). In particular, opinions relating to the robot’s sensors and privacy differed greatly among participants. Whereas some of the participants expressed concerns in this area, as described in category 3, others did not see any problems here: "In principle, it is completely unproblematic, since the camera only captures a moment in time" (PN 10, FG 2).

Patients especially very rarely raised concerns about the robot’s use. On the subject of fear or insecurity, as discussed in category 1, one patient explained she would not be afraid of the robot: "I'm not afraid because when a person is there too, everything is okay" (Patient (P) 2). In the matter of human relationships (see category 4), one nurse stated “I think it can't disturb a relationship […] because this robot does not replace the nursing and the human touch during care" (PN 16, FG 3]”. One patient even came up with a possible positive effect of the robot, here: "And the robot is never in a bad mood" (P3).

#### Prioritising of ethical issues

At the end of the focus group discussion, the moderators asked the participating nurses to name up to three aspects of the discussion that they considered most important. Nurses named issues of safety and effort in use most frequently. Numerous other prioritised issues were the loss of functional patient resources and deterrence due to the robot’s appearance. In this regard, the participants suggested giving the robot a name and making it look less industrial. On the other hand, issues concerning personal privacy at the relationship level or staffing ratio were rarely named as being most important. The results of the voting are summarized and sorted by frequency in Table 3.

**Table 3:** Most important aspects (nurses' perspective)

Aspect mentioned as most important	Frequency of mentions
Safety	10
Efficiency/ effort in use	8
Deterrence due to robot’s appearance	5
Loss of functional patient resources	4
Costs	2
Personal privacy	1
Relationship level	1
Staffing ratio	1
Small number of available robots	1

### Identified ethical risks and requirements

The complete table with all identified risks, mitigating requirements and defined validation/ verification methods is presented in additional file 3. The structure of ethical issues is guided by the categories from the analysis of focus groups and individual interviews. Risks that arose in focus groups or individual interviews are included in the table.

There was a large overlap between risks identified in focus groups/ individual interviews and in the literature. For example, the issues of staffing ratio [15, 16], reduction of human contact [17] and liability [18] have also been emphasized in the literature. A risk that did not arise in the focus groups or individual interviews but did so in the literature was that of an incomplete representation of phenomena in the population, leading to errors in use for persons with certain characteristics [6]. For the project this could specifically mean that detecting the position of a patient’s leg leads to errors when the patient’s skin colour is black. Further risks regarding robots in the literature revolve, for instance, around machine learning and lack of transparency concerning robotic actions [6, 10, 19] or the misuse of robots [6, 10]. Additional risks contributed by members of the research team were, among others, the fear that holistic care might get pushed into the background and the idea that efficiency could take precedence. Furthermore, environmental issues were not discussed in the focus groups or individual interviews. Members of the research team therefore added the risks that a partial defect could lead to total uselessness and that resources used for the conception and use of the robot might cause damage to the environment.

Mitigating requirements were assigned to ethical risks. For example, to mitigate the risk of deception an anthropomorphic design should be limited, perhaps by means of a functional design that is oriented towards the technical properties of the robot [20]. Regarding the risk of errors in use for persons with specific characteristics, data for the adjustment of the robot should be inclusive and represent different population groups [19]. The specific requirement for the project is therefore that the robot should be able to process male and female voices, different accents and skin colours equally well. With regard to risks of inappropriate control by the robot, it is suggested to limit robotic self-learning without human supervision [10] and to keep algorithms verifiable [19]. To mitigate problems in the field of personal privacy, there are legal requirements that need to be respected. Accordingly, personal data should only be processed with the consent of those concerned [21]. Furthermore, the principle of data minimization applies. Therefore, only data that is necessary for the fulfilment of the purpose should be collected, and should be deleted immediately afterwards [21]. For further requirements, see additional file 3.

To define validation or verification methods, hard-/software verification was assigned to requirements when these addressed the functions or design of the robot, such as limiting anthropomorphic appearance or that sensors should be able to deal with different human characteristics. Social assessment was chosen when the corresponding risk or mitigation was about social outcomes such as the reputation of patients or nurses, and legal assessment when aspects of law must be respected, as in the field of personal data or liability. User validation was defined as a validation method when a personal opinion by users is needed, e.g. to answer the question how persons in need of care experience the treatment with the robot.

## Discussion

Ethical risks and requirements have been defined in the context of dignity, autonomy, privacy, human relationships and safety in the project. Numerous risks could be identified through focus groups with professional nurses, in which participants feared most issues relating to safety and that the robot would not lead to relief but to more workload eventually. Despite the focus on possible negative consequences of the robot under investigation in the future, it became clear that nurses rated some of the discussed risks as unproblematic or rather saw advantages of the robot’s use in the future. In the individual interviews, relatives and patients only seldom raised concerns about using the robot in the future.

For the overall process of implementing ethical aspects, the BS 8611 served as a guiding framework. We experienced the BS 8611 as helpful in providing a structured procedure that goes beyond the identification of risks alone. However, it contains substantive rather than concrete methodological suggestions for the implementation of individual steps. Also, the way how to involve users is up to the applying researchers, as is also remarked by Stahl & Coeckelbergh [9]. Non-engineers might feel it unfamiliar to work with the BS 8611, but for the PfleKoRo project the described procedure provided opportunities to link ethical work closely to the work of engineers. It therefore seemed promising not just to continue accompanying research, but to be able to influence the development of the system. So far, in line with the appraisal of Stahl & Coeckelbergh, we generally consider the BS 8611 as suitable for realizing the ideas of RRI and for applying it in the early research and development stages. However, when applying the BS 8611 one should be aware that the BS 8611 starts with drawing a negative perspective and one should ask the question “What should be avoided?” with the focus on risks. It is therefore less able to draw desirable future scenarios and identify chances.

To involve people potentially affected by the robot under investigation, we chose individual interviews and focus groups to identify ethical risks from the perspective of nurses, patients and relatives.

Only few concerns about the robot emerged in individual interviews, especially with patients. One possible explanation could be an uncritical attitude towards robotics in health care in general. At the beginning of the interviews, which was not part of the analysis, the participants differed in their response when the interviewer asked the following introductory question: “What was the first thought that came to your mind when you heard that a team wanted to develop a robot for nursing care?”. One of the relatives and three of the patients mentioned exclusively positive or neutral comments. Other participants also expressed critical thoughts at this point. Existing research on the attitudes of patients and older people towards robots for health care also come to inconsistent conclusions here. The authors of one review found that older people had more positive than negative attitudes towards robots in health care [22]. However, an interview study revealed that patients prefer human interaction in care on the one hand, but also advocate the use of robots for some caregiving tasks [23]. Quantitative surveys in Germany showed that 40% of people over the age of 70 years fundamentally rejected assistance by robots, whereas 82% of older people could imagine the use of robots as long as this allows them to live at home longer [24]. Both the statements of participants in this study as well as those in existing research do not suggest a uniformly positive attitude towards robots in health care. Maybe a larger number of interviews and a more comprehensive interview frame for ethical concerns alone would have revealed more results.

In contrast to the individual interviews, a large number of ethical risks could be identified in the focus groups. Although the digital realization required special preparation and moderation, it proved beneficial in other matters. The digital format allowed bringing participants from different locations and working settings together, which enriched the discussion. Another challenge for both the focus groups and individual interviews was the early stage of the robot’s development and the fact that the participants had no experience with the robot. This required a high level of anticipation and imagination from the participants. For the focus groups, it proved helpful to provide a sufficient time frame, to present a possible application scenario and to have a preparation phase, as described in the methods section. The focus groups implemented in this way proved to be suitable for involving users in the anticipation of ethical risks in relation to the robot’s early developmental stage, as aimed at by the idea of RRI. Apart from the number of identified risks in focus groups, it became apparent that the participants viewed ethical issues very differently. Although it is not one of this study’s objectives, we hope to gain a more systematic overview of how potential users rate ethical risks in relation to the robot at a current state of development, which will be required for the forthcoming evaluation of a prototype.

Some ethical issues not raised in the interviews and focus groups but relevant for the robot under investigation could be added from the literature and by members of the research team. By using various sources, the most comprehensive results possible could be achieved. At the same time, it was possible to gain insights into the perspectives of those potentially affected by the robot. The results thus expand the pool of ethical risks and requirements that are relevant for a robot for nursing care and suggest which ones should be especially considered from the perspective of nurses, patients and relatives.

### Methodological considerations

Due to the SARS-CoV-2 pandemic, access to patients and relatives was difficult and only a small sample size was recruited for the individual interviews. In order to inconvenience hospitalized patients and relatives as little as possible, the interviews only touched briefly on the topic of ethical issues. Therefore, there was only limited room for the perspectives of patients and relatives. We hope to gain deeper insights into patient and relative ratings of ethical risks during the forthcoming evaluation of the prototype.

Likewise, to avoid extra inconvenience for participants, especially in the context of SARS-CoV-2, transcripts and findings from the interviews and focus groups were not returned to participants for their feedback. Instead, a digital whiteboard was used during focus groups to take notes of the participants’ contributions. The whiteboard was visible for all participants during the whole discussion, thus providing the opportunity for direct feedback.

Convenience sampling was used for recruitment of participants and data saturation was not the criterion for stopping recruitment. Nevertheless, the first two focus groups provided plenty of data already with occasional supplements from the last focus group. In the individual interviews too, the only aspect mentioned many times was that of reduction in staffing ratio, and only seldom was something added during the course of the interviews. This observation coincides with meta-research e.g. by Hennink et al. [25], showing that more than 80% of the codes could be identified in two focus groups and more groups provided only little additional benefit.

The participants in the focus groups had a wide range of age and working experience and came from different backgrounds. To reduce possible issues of competence, the moderator made sure that all the participants were involved in the discussions and encouraged them to share their thoughts, even if they differed from the views of other participants. Apart from that, the heterogeneity within the groups enriched the discussion and promoted exploring different views.

To reduce the possible influence of a single researcher, two researchers analysed the data from the focus groups and interviews together. In addition, the professional nurse in the research team participated in analysing the data and validated the transfer of identified risks in focus groups and interviews to the list of ethical risks. For the overall procedure, multiple sources of data in form of focus groups, interviews, literature and expert guidance were involved to compensate limitations of one single method.

## Conclusions

This study revealed several ethical risks and requirements in the context of dignity, autonomy, privacy, human relationships and safety related to a robot for nursing care. In particular, it shed light on the perspective of people potentially affected by the technology. Professional nurses feared most risks related to safety and that the robot would lead to more work load instead of relief, whereas patients and relatives more often raised the issue of the staffing ratio. Along with those concerns, participants made many uncritical and positive comments that suggest their general open-mindedness for the introduced robot. Further risks like discrimination of users with certain characteristics and requirements could be added from existing literature. As the different sources lead to partially different ethical issues and main points of interest, it is advisable to involve multiple perspectives and potentially affected people in particular when investigating ethical implications of a robot for nursing care.

## Supplementary Information

**Additional file 1.** Application scenario with the robot for focus groups.

**Additional file 2.** Code book.

**Additional file 3. **Ethical risks and requirements.

## Data Availability

The datasets generated and analysed during the current study are not publicly available since the participants did not consent to the publication of the transcripts for all purposes but are available from the corresponding author on reasonable request.

## Abbreviations

FG: Focus group
P: patient
PN: professional nurse
R: relative
RRI: Responsible Research and Innovation

## References

[1] Fehling P, Dassen T (2017). Motive und Hürden bei der Etablierung technischer Assistenzsysteme in Pflegeheimen: eine qualitative Studie [Motives and obstacles in establishing technical assistance systems in nursing homes: a qualitative study]. Klinische Pflegeforschung.

[2] Merda M, Schmidt K, Kähler B. Pflege 4.0- Einsatz moderner Technologien aus der Sicht professionell Pflegender: Forschungsbericht [Use of modern technologies from the perspective of professional caregivers: Research report]. 2017. https://www.bgw-online.de/resource/blob/20346/e735030f6178101cf2ea9fa14e1bc063/bgw09-14-002-pflege-4-0-einsatz-moderner-technologien-data.pdf. Accessed 24 Nov 2021.

[3] Rantanen T, Lehto P, Vuorinen P, Coco K (2018). The adoption of care robots in home care-A survey on the attitudes of Finnish home care personnel. J Clin Nurs.

[4] Becker H, Scheermesser M, Früh M, Treusch Y, Auerbach H, Hüppi RA, Meier F. Robotik in der Gesundheitsversorgung: Hoffnungen, Befürchtungen und Akzeptanz aus Sicht der Nutzerinnen und Nutzer [Robotics in health care: hopes, fears and acceptance from the users‘ point of view]. In: Bendel O, editor. Pflegeroboter. Wiesbaden: Springer Gabler; 2018. 10.1007/978-3-658-22698-5_13.

[5] Sharkey N, Sharkey A (2012). The eldercare factory. Gerontology.

[6] Philip Jansen, Philip Brey, Alice Fox, Jonne Maas, Bradley Hillas, Nils Wagner, et al. SIENNA D4.4: Ethical Analysis of AI and Robotics Technologies (V1.1). 2020. https://zenodo.org/record/4068083#.YZ4ITNDMKUk. Accessed 24 Nov 2021.

[7] Stilgoe J, Owen R, Macnaghten P (2013). Developing a framework for responsible innovation. Res Policy.

[8] Burget M, Bardone E, Pedaste M (2017). Definitions and conceptual dimensions of responsible research and innovation: a literature review. Sci Eng Ethics.

[9] Stahl BC, Coeckelbergh M (2016). Ethics of healthcare robotics: towards responsible research and innovation. Robot Auton Syst.

[10] BSI (2016). Robots and robotic devices: Guide to the ethical design and application of robots and robotic systems (BS 8611:2016) 2016: BSI Standards Limited.

[11] Feldhusen J, Grote KH, editors. Pahl/Beitz Konstruktionslehre: Methoden und Anwendung erfolgreicher Produktentwicklung [Pahl/ Beitz construction theory: methods and application of successful product development]. 8th ed. Springer Vieweg: Berlin, Heidelberg; 2013.

[12] Hofmann B, Droste S, Oortwijn W, Cleemput I, Sacchini D (2014). Harmonization of ethics in health technology assessment: a revision of the Socratic approach. Int J Technol Assess Health Care.

[13] EUnetHTA Joint Action 2, Work Package 8. HTA Core Model®version 3.0 (pdf). 2016. www.htacoremodel.info/BrowseModel.aspx. Accessed 23 Nov 2021.

[14] Kuckartz U (2018). Qualitative Inhaltsanalyse. Methoden, Praxis, Computerunterstützung [Qualitative content analysis. Methods, practice, computer support].

[15] Manzeschke A. MEESTAR: Ein Modell angewandter Ethik im Bereich assistiver Technologien [MEESTAR: A model of applied ethics in assistive technologies]. In: Weber K, editor. Technisierung des Alltags. Beitrag für ein gutes Leben? Stuttgart: Steiner; 2015.

[16] Johansson-Pajala RM, Gustafsson C. Significant challenges when introducing care robots in Swedish elder care. Disabil Rehabil Assist Technol. 2020:1–11. 10.1080/17483107.2020.1773549. 10.1080/17483107.2020.1773549 32538206

[17] Remmers H, Bendel O (2018). Pflegeroboter: Analyse und Bewertung aus Sicht pflegerischen Handelns und ethischer Anforderungen [Care robots: Analysis and evaluation from the point of view of care activities and ethical requirements]. Pflegeroboter.

[18] Kehl C. Bericht des Ausschusses für Bildung, Forschung und Technikfolgenabschätzung (18. Ausschuss) gemäß § 56a der Geschäftsordnung: Technikfolgenabschätzung (TA). Robotik und assistive Neurotechnologien in der Pflege- gesellschaftliche Herausforderungen [Report of the Committee on Education, Research and Technology Assessment (18th Committee) in accordance with Section 56a of the rules of procedure: Technology assessment (TA). Robotics and assistive neurotechnologies in nursing care- societal challenges]. 2018. https://dip.bundestag.de/vorgang/bericht-des-ausschusses-f%C3%BCr-bildung-forschung-und-technikfolgenabsch%C3%A4tzung-18-ausschuss/236579. Accessed 24 Nov 2021.

[19] High-Level Expert Group on Artificial Intelligence. Ethics Guidelines for Trustworthy AI. 2019. https://www.aepd.es/sites/default/files/2019-12/ai-ethics-guidelines.pdf. Accessed 23 Nov 2021.

[20] Onnasch L, Jürgensohn T, Remmers P, Asmuth C. Ethische und soziologische Aspekte der Mensch-Roboter-Interaktion [Ethical and sociological aspects of human-robot interaction]. 1st ed. Dortmund: Bundesanstalt für Arbeitsschutz und Arbeitsmedizin; 2019.

[21] Steckler B, Krempel E, Gransche B, Manzeschke A (2020). “Privacy by Design” im Dialog von Recht und Technik [“Privacy by design” in the dialogue between law and technology]. Das geteilte Ganze: Horizonte Integrierter Forschung für künftige Mensch-Technik-Verhältnisse.

[22] Savela N, Turja T, Oksanen A (2018). Social acceptance of robots in different occupational fields: a systematic literature review. Int J of Soc Robotics.

[23] Vallès-Peris N, Barat-Auleda O, Domènech M (2021). Robots in Healthcare? What Patients Say. Int J Environ Res Public Health.

[24] Rebitschek FG, Wagner GG. Akzeptanz von assistiven Robotern im Pflege- und Gesundheitsbereich : Repräsentative Daten zeichnen ein klares Bild für Deutschland. [Acceptance of assistive robots in the field of nursing and healthcare: representative data show a clear picture for Germany]. Z Gerontol Geriatr. 2020;53:637–43. 10.1007/s00391-020-01780-9. 10.1007/s00391-020-01780-9 32945928

[25] Hennink MM, Kaiser BN, Weber MB (2019). What influences saturation? Estimating sample sizes in Focus Group Research. Qual Health Res.

