# Supplementary material for: Implementing ethical aspects in the development of a robotic system for nursing care: a qualitative approach
Source: BMC Nurs. 2022 Jul 8;21:180. doi: 10.1186/s12912-022-00959-2 (PMC9263068; doi:10.1186/s12912-022-00959-2)
Supplement: Supplementary file 1 — Additional file 1. Application scenario with the robot for focus groups. [file 12912_2022_959_MOESM1_ESM.pdf]

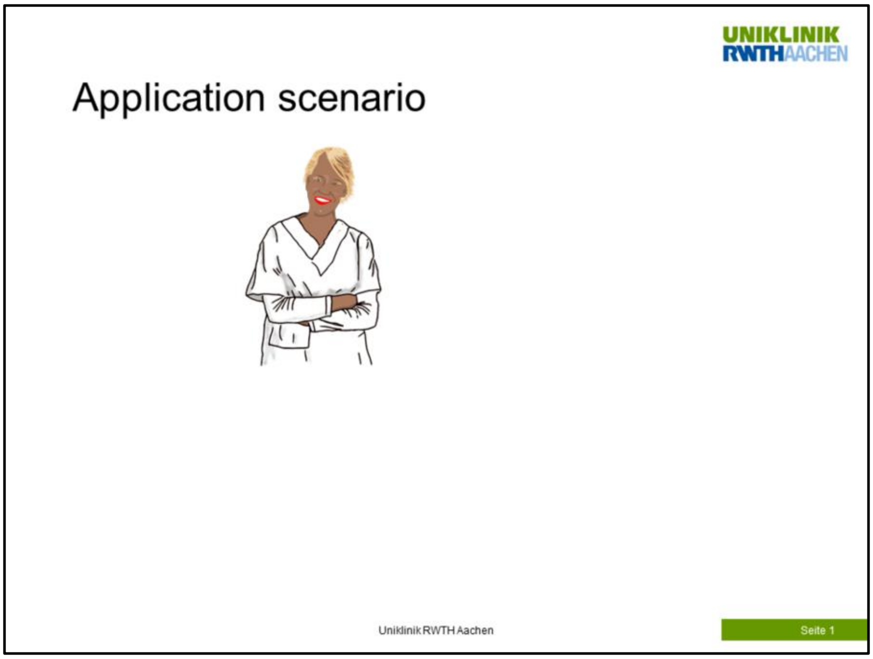

This is Sarah. Sarah is a professional nurse and currently on duty caring for…

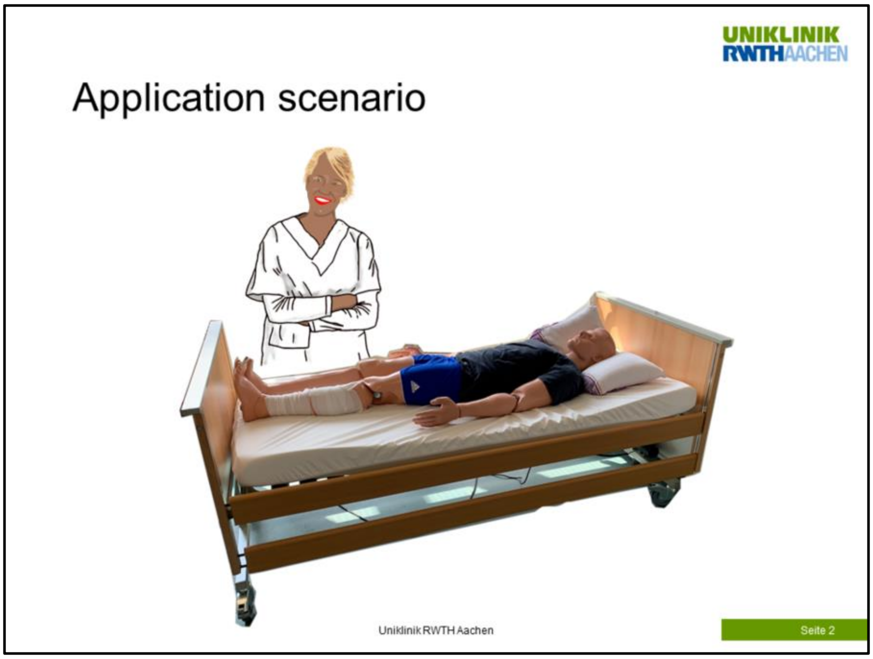

…her patient, Mr. M. Due to severe physical restrictions, Mr M. can hardly help with nursing measures himself. So Sarah needs support.

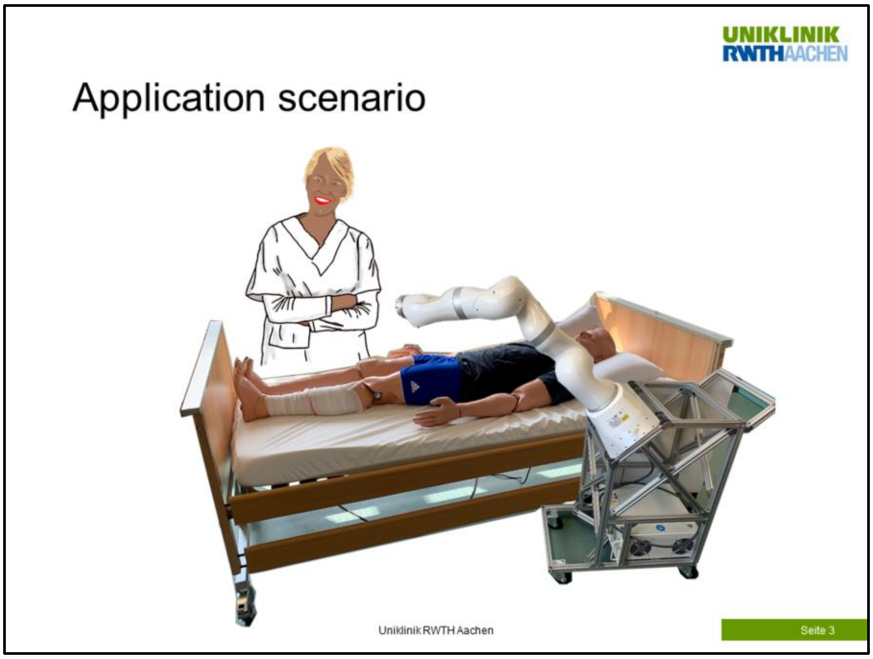

Therefore, she is going to use the PfleKo robot today.

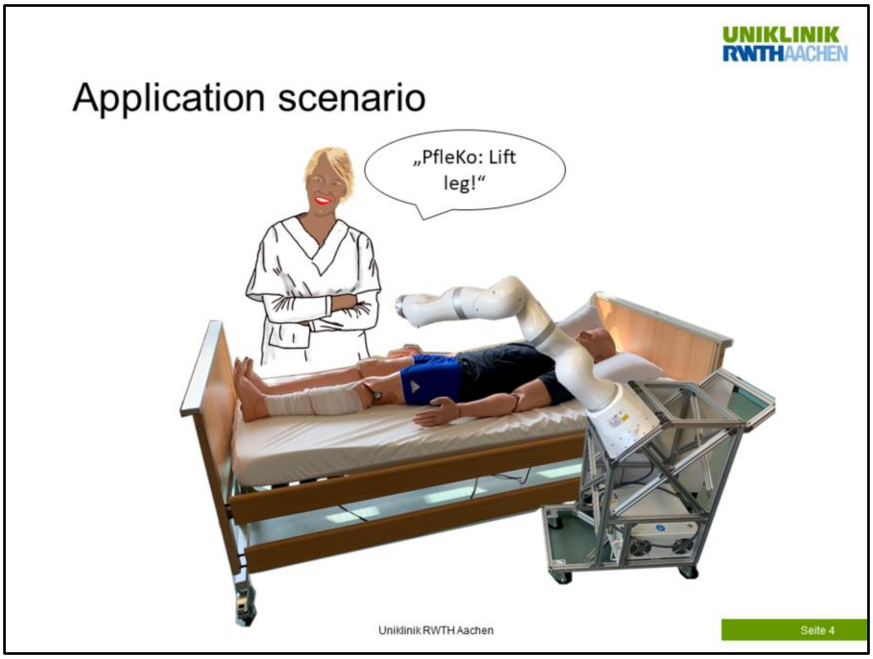

Sarah would like to replace the wound bandage on Mr. M's lower leg. To do this, she says: “PfleKo: Lift leg!”

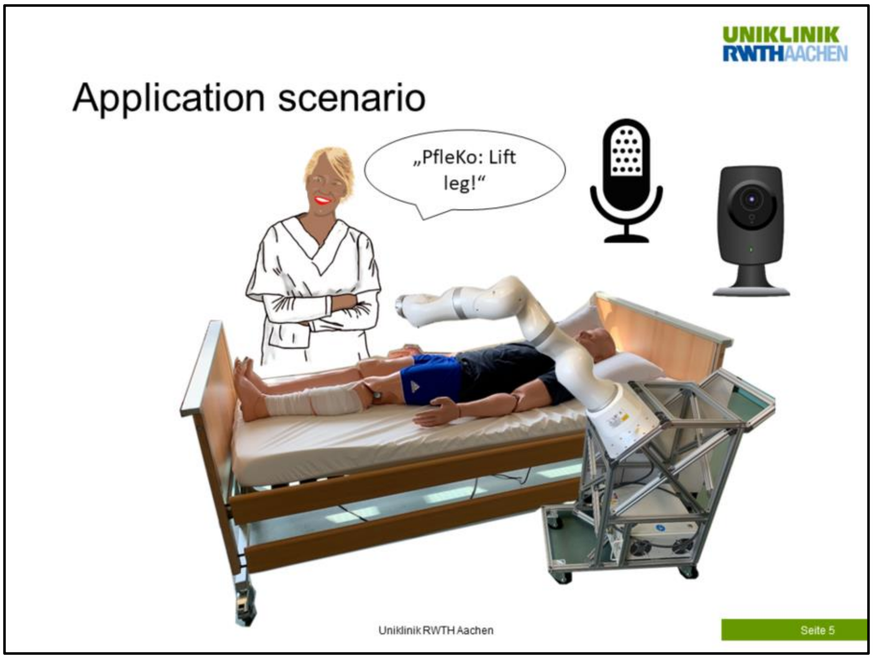

The robot processes the command with the help of a microphone. Using a camera, the robot finds the right place to hold Mr. M‘s leg.

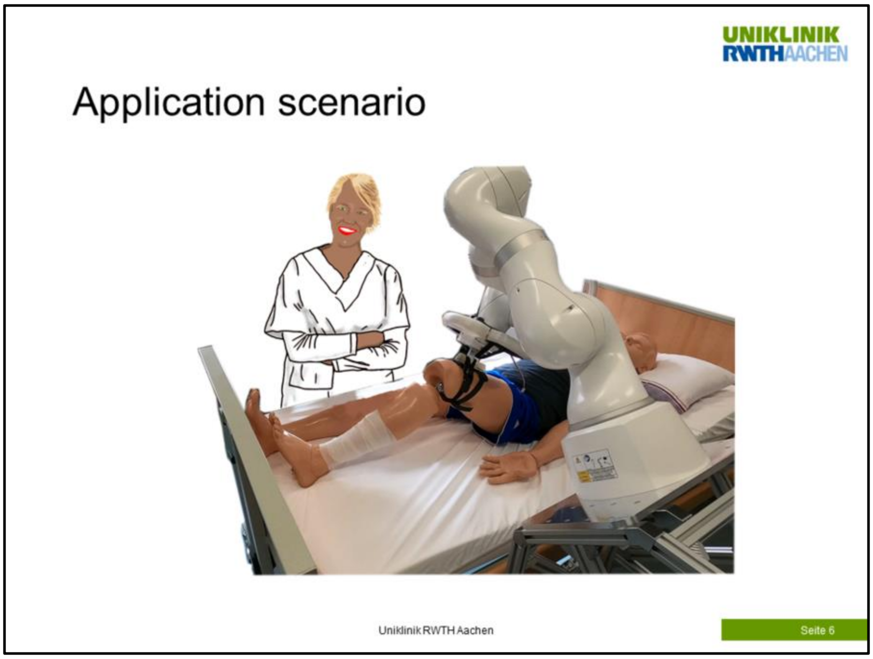

While the robot is holding Mr. M's leg, Sarah can treat the wounds and replace the bandage.
